# Pregnancy-adapted YEARS Algorithm: A Retrospective Analysis

**DOI:** 10.5811/westjem.60626

**Published:** 2023-12-08

**Authors:** Alden Mileto, Gina Rossi, Benjamin Krouse, Robert Rinaldi, Julia Ma, Keith Willner, David Lisbon

**Affiliations:** * Geisinger Commonwealth School of Medicine, Scranton, Pennsylvania; † Geisinger Wyoming Valley Health Center, Department of Emergency Medicine, Wilkes-Barre, Pennsylvania

## Abstract

**Introduction:**

Pulmonary embolism (PE) is an imperative diagnosis to make given its associated morbidity. There is no current consensus in the initial workup of pregnant patients suspected of a PE. Prospective studies have been conducted in Europe using a pregnancy-adapted YEARS algorithm, which showed safe reductions in computed tomography pulmonary angiography (CTPA) imaging in pregnant patients suspected of PE. Our objective in this study was 1) to measure the potential avoidance of CTPA use in pregnant patients if the pregnancy-adapted YEARS algorithm had been applied and 2) to serve as an external validation study of the use of this algorithm in the United States.

**Methods:**

This study was a single-system retrospective chart analysis. Criteria for inclusion in the cohort consisted of keywords: pregnant; older than 18; chief complaints of shortness of breath, chest pain, tachycardia, hemoptysis, deep vein thromboembolism (DVT), and D-dimer—from January 1, 2019– May 31,2022. We then analyzed this cohort retrospectively using the pregnancy-adapted YEARS algorithm, which includes clinical signs of a DVT, hemoptysis, and PE as the most likely diagnosis with a D-dimer assay. Patients within the cohort were then subdivided into two categories: aligned with the YEARS algorithm, or not aligned with the YEARS algorithm. Patients who did not receive a CTPA were analyzed for a subsequent diagnosis of a PE or DVT within 30 days.

**Results:**

A total of 74 pregnant patients were included in this study. There was a PE prevalence of 2.7% (two patients). Of the 36 patients who did not require imaging by the algorithm, seven CTPA were performed. Of the patients who did not receive an initial CTPA, zero were diagnosed with PE or DVT within a 30-day follow-up. In total, 85.1% of all the patients in this study were treated in concordance with the pregnancy-adapted YEARS algorithm.

**Conclusion:**

The use of the pregnancy-adapted YEARS algorithm could have resulted in decreased utilization of CTPA in the workup of PE in pregnant patients, and the algorithm showed similar reductions compared to prospective studies done in Europe. The pregnancy-adapted YEARS algorithm was also shown to be similar to the clinical rationale used by clinicians in the evaluation of pregnant patients, which indicates its potential for widespread acceptance into clinical practice.

Population Health Research Capsule What do we already know about this issue?

*Pulmonary embolism is challenging to diagnose in pregnant patients. In European studies the pregnancy-adapted YEARS algorithm has shown promise in simplifying this diagnosis.*

What was the research question?

*We investigated the reduction in computed tomography (CT) achieved by applying the YEARS algorithm to pregnant patients in two US hospitals.*

What was the major finding of the study?

*In our 74-patient sample, use of the YEARS algorithm could have safely avoided seven CTs (19.4% reduction).*

How does this improve population health?

*Adoption of the pregnancy-adapted YEARS algorithm could safely reduce CT imaging in pregnant patients, reducing their radiation exposure and streamlining ED workup.*

## INTRODUCTION

One of the challenges the emergency physician faces is the prompt diagnosis of pulmonary embolism (PE) in pregnant patients. Pulmonary embolism remains a significant cause of maternal mortality.^1^ ^–^ ^3^ Studies show that approximately 9% of pregnancy-related deaths in the United States are due to a PE.^2^ Causes include physiologic changes in pregnancy that induce a hypercoagulable state, which predisposes patients to venous thromboembolism (VTE).^4^ ^–^ ^6^ The normal physiologic changes in pregnancy substantially overlap with the clinical signs and symptoms of PE, which further complicates PE workups within this population. D-dimer testing, widely used in non-pregnant patients, is controversial in pregnancy because its accuracy varies by trimester.^3^ ^,^ ^7^ Proposals for age-adjusted or trimester-adjusted cut-off values have been or are currently being considered.^8^ ^–^ ^10^

Reports show that the prevalence of PE in pregnant patients undergoing diagnostic workup in the emergency department (ED) is approximately 3.7%, whereas nonpregnant patients of childbearing age showing a PE prevalence of 6.0%.^11^ Diagnostic workup, such as computed tomography pulmonary angiography (CTPA) or a V/Q scan, increases costs and evaluation times. These scans expose the fetus to radiation. Analyses have shown a 121% increase in radiologic examinations in pregnant women from the years 1997–2006.^12^ While radiation poses potential teratogenic effects, these effects are dose-dependent and vary based on gestational age. Radiation exposures greater than 500 milligray (mGy) cause fetal damage, and exposure to less than 50 mGy has not been associated with differences in pregnancy outcomes.^13^ While CTPA is associated with radiation exposure of <5 mGy, given the complexities of the effects of exposure based on gestational age and other radiation exposure during the pregnancy, it is recommended that the potential benefit of the radiologic study be weighed against the radiation exposure to the fetus.^12^ ^,^ ^13^ Multiple criteria have been developed to aid clinicians in quickly assessing and diagnosing PE including Wells, PE rule-out criteria (PERC), and YEARS criteria. However, these criteria were originally developed excluding pregnant patients from their studies, which has resulted in a lack of consensus on PE workup in pregnant individuals.^14^

**Figure 1.**
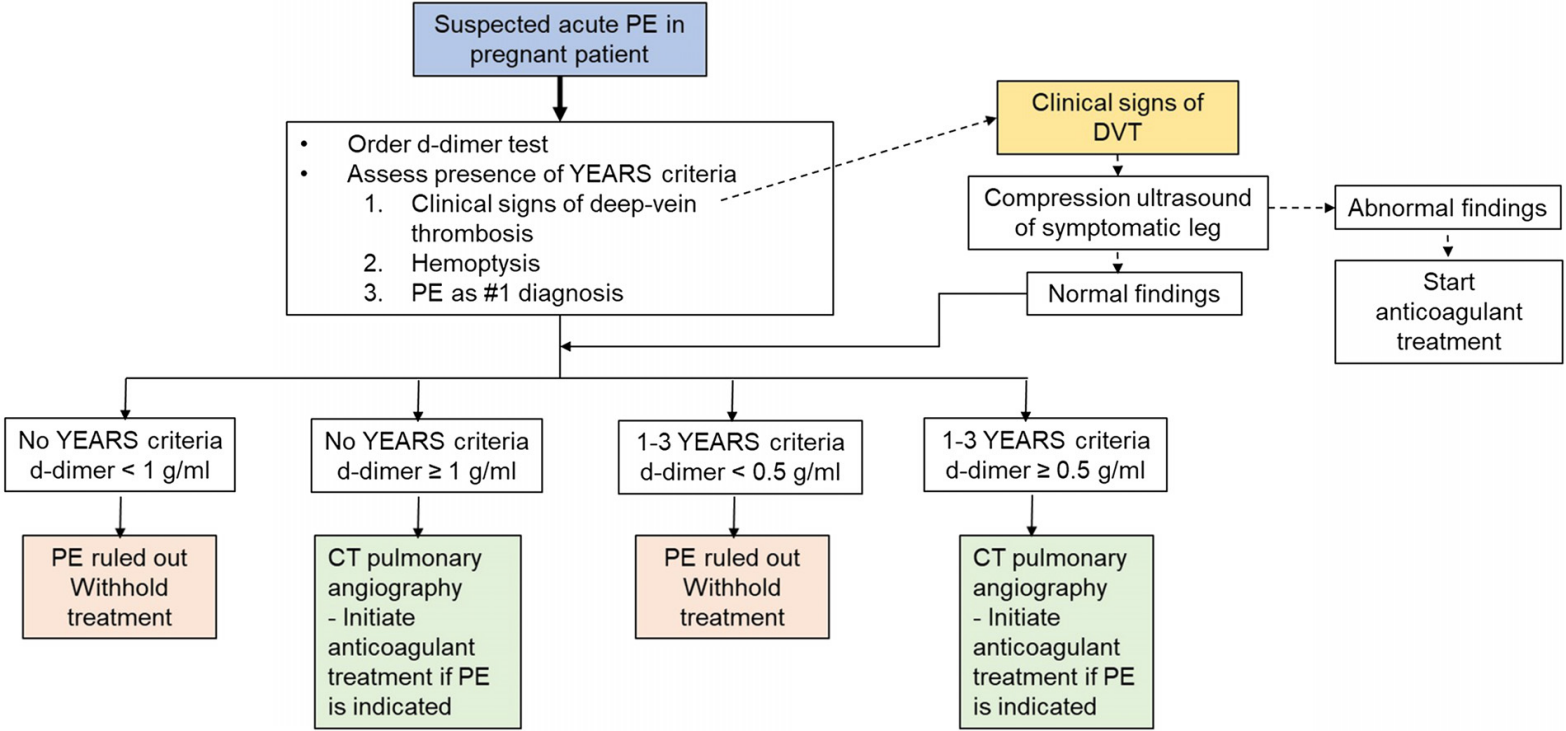
Pregnancy-adapted YEARS algorithm for management of suspected acute pulmonary embolism in pregnant patients. *DVT*, deep-vein thrombosis; *PE*, pulmonary embolism; *YEARS*, diagnostic algorithm for pulmonary embolism; *CT*, computed tomography; *g/mL*, grams per milliliter.

Recent studies have demonstrated greater efficacy of the YEARS criteria, in comparison to the traditionally used PERC and Wells criteria.^15^ ^–^ ^17^ In 2019, an international study aimed to clinically evaluate PE in pregnant patients using a pregnancy-adapted YEARS algorithm.^14^ Their conclusion was that a pregnancy-adapted YEARS algorithm proved viable in ruling out a PE without serious adverse consequences. The pregnancy-adapted YEARS algorithm is summarized in Figure 1.

Prior prospective studies applying the pregnancy-adapted YEARS algorithm took place in Europe.^14^ ^,^ ^18^ Additionally, another study reviewed the prevalence of PE in North America and Europe in non-pregnant patients. The prevalence of patients tested for PE in Europe was 23% compared to 8% in North America. This study also reported both a lower rate of CTPA utilization (38% vs 60%) and a lower diagnostic yield from CTPA (13% vs 29%) in North America.^19^ The objective of our study was to measure the potential avoidance of CTPA in pregnant patients being evaluated for a PE if the pregnancy-adapted YEARS algorithm had been applied and to serve as an external validation study of the use of this algorithm in the US.

## METHODS

### Study Design

This study was a retrospective chart analysis conducted on visits from January 1, 2019–May 31, 2022, spanning one Level I trauma center/tertiary care center and one urban community hospital in Pennsylvania. The cohort included pregnant patients ≥18 years of age who presented to the ED with chief complaints consistent with a suspected PE—shortness of breath, chest pain, tachycardia, hemoptysis, and clinical signs of deep vein thromboembolism (DVT). For the robustness of the dataset our search strategy also included pregnant patients for whom a D-dimer had been ordered. We excluded patients who did not receive a D-dimer test as part of their clinical workup. We also excluded patients who were worked up for a PE outside their pregnancy period. Procedures and protocols were approved by the institutional review board.

### Procedures

Patients for this study were gathered by an initial search strategy that used the SlicerDicer feature in the Epic electronic health record (Epic Systems Corporation, Verona, WI). SlicerDicer is a validated tool within Epic that allows for the selection of patients given certain inclusion and exclusion data.^20^ Trained medical student research assistants (RA) extracted patient data via retrospective chart review. The RAs were initially blinded to the study outcome. The two senior authors (KW, DL), both board-certified emergency physicians, reviewed a random sampling of each abstractor’s charts for accuracy. Each chart was then tabulated by chief complaint and subsequent findings according to the YEARS algorithm summarized in Figure 1, regardless of whether the algorithm was used in patient workup. Any questionable cases were reviewed once more by an attending physician.

Clinical signs of a DVT included documented clinician suspicion of a DVT or documented unilateral or bilateral leg swelling, warmth, pain, or discoloration. Hemoptysis was deemed present if the patient reported hemoptysis during the visit, within 24 hours of a visit, or was determined by the evaluating clinician to be relevant. Pulmonary embolism as the most likely diagnosis was determined through thorough evaluation of health records. A detailed methodology of how “PE most or equally likely diagnosis” was determined is elucidated in the supplemental attachment. Any disagreement in the determination of PE as the “most or equally likely” diagnosis triggered review by a senior author and was resolved by consensus. The RAs evaluated charts independently, and ultimately all charts adjudicated as “PE most or equally likely diagnosis” were discussed by both senior authors; therefore, we did not calculate a kappa statistic. Missing historical or clinical exam findings were treated as absent.

If the CTPA showed a new filling defect in any pulmonary artery, PE was assumed to be present.^21^ If the result of a compression ultrasonography showed noncompressibility of a proximal vein, a DVT was assumed to be present.^19^ Patients were then further categorized as nonconcordant or concordant with the pregnancy-adapted YEARS algorithm (Figure 1).

Patients who did not receive a CTPA were assessed within a 30-day follow-up period. These visits included subsequent appointments in which the previous ED visit was addressed. Further analysis at the follow-ups included workup for suspected VTE, PE, or an additional ED visit as recommended by the treating clinician. All follow-up visits were within 30 days from the initial ED encounter for PE workup. Additionally, all patients in the study completed their pregnancy in the health system.

### Analysis

We used Excel (Microsoft Corporation, Redmond, WA) to perform fundamental statistical calculations. To maintain data integrity and ensure ongoing data accuracy we implemented regular quality control procedures, including periodic reviews and spot-checking. This involved random sampling of entered data for extrinsic verification. We did not use data software to collect data.

## RESULTS

A total of 323 patients were found via the initial search strategy. After removing duplicates and patients who were not pregnant and did not have a D-dimer test performed, 67 cases remained. These cases were cross-referenced with the system’s internal radiology database, which records pregnancy status of all patients who received ionizing radiation, yielding an additional seven cases for analysis. During the study period, 74 patients were evaluated for PE. The patients were 19–38 years old (mean age 27.85). The highest percentage (41.9%) of patients were in the third trimester of pregnancy at the time of their evaluation. The presenting complaints of the patients reviewed are summarized in Table 1.

**Table 1.** Pregnancy demographics and chief complaints of patients suspected of pulmonary embolism.

	Total (%)
Population demographics	
Age Range	19–38 years
Mean Age	27.85 years
Age Standard Deviation	5.04 years
Trimester	
1st Trimester	16 (21.6%)
2nd Trimester	27 (36.4%)
3rd Trimester	31 (41.9%)
Patient Presentation	
Shortness of breath only	29 (39.2%)
Chest pain only	21 (28.4%)
Chest pain and shortness of breath	13 (17.6%)
Cold symptoms/COVID-19 symptoms	4 (5.4%)
Other	7 (9.5%)

Seven of the 74 patients reviewed did not have D-dimer testing completed, and thus were excluded from the analysis to determine the effectiveness of the pregnancy-adapted YEARS algorithm. Five of the excluded patients met at least one YEARS criteria, and two of those five patients were found to have a PE. These two patients comprise the 2.7% prevalence of PE in our study cohort. A breakdown of the range of D-dimer levels is represented in Table 2. Among the 67 patients included in the analysis, 47 patients (70.15%) met no YEARS criteria, and 20 patients (29.85%) met one or more YEARS criteria. Eighteen patients (90%) met the criteria of PE being considered the number one diagnosis, one patient (5%) had unilateral leg swelling, and one patient (5%) had both hemoptysis and PE considered as the number one diagnosis.

**Table 2.** Breakdown of number of patients within certain ranges of D-dimer levels stratified by YEARS criteria met.^*^

D-dimer level	Number of patients
0 YEARS Criteria	
<0.5 mg/L	15
0.5–1.0 mg/L	20
>1 mg/L	12
≥1 YEARS Criteria	
<0.5 mg/L	1
>0.5 mg/L	19

* Below the standard cutoff of 0.5 mg/L, between the standard cutoff and the pregnancy-adapted YEARS algorithm level of 1.0 mg/L with no YEARS criteria, and above the algorithm’s cutoff level (0.5 mg/L or 1.0 mg/L), depending on whether YEARS criteria were met.

*mg/L*, milligrams per lliter.

Among the 47 patients who did not meet any of the three YEARS criteria, 35 (74.47%) had a D-dimer below the threshold of 1.0 milligrams per liter (mg/L), and 12 (25.53%) had a D-dimer greater than 1.0 mg/L. Of those 35 patients who should not have undergone CTPA based on the pregnancy-adapted YEARS algorithm, seven (20%) had a CTPA performed. These seven patients represent the patients who could have avoided radiation exposure with application of the YEARS-adapted algorithm. Four of these patients had D-dimer levels between 0.5–1.0 mg/L, and three patients had a D-dimer level <0.5 mg/L. None of these seven patients were found to have a PE on imaging. Among the 28 patients who did not have a CTPA performed, 24 patients (85.71%) had a follow-up evaluation in the health system within 30 days, and none were found to have a VTE diagnosed. Four patients (14.29%) did not have a follow-up visit documented within 30 days of their PE workup in the ED. Of note, of the 28 patients who did not have a CTPA performed, 16 (67.14%) had D-dimer levels between 0.5–1.0 mg/L.

Of the 12 patients who met zero YEARS criteria and had a D-dimer of greater than 1.0 mg/L, 10 (83.33%) had a CTPA performed, all of which showed no PE. Two (16.67%) of these 12 patients did not have a CTPA performed. One of these did not have a follow-up visit documented within 30 days of their PE workup in the ED. However, this patient had no diagnosis of VTE or new anticoagulant medication listed on admission to labor and delivery.

Of the 20 patients with one or more YEARS criteria, 19 (95%) had a D-dimer >0.5 mg/L, and one patient (5%) had a D-dimer of <0.5 mg/L. The patient with a D-dimer of <0.5 mg/L did not have a CTPA performed and had no VTE at 30-day follow-up. Of the 19 patients with D-dimer levels of >0.5 mg/L, 17 (89.47%) had CTPA imaging performed and one (5.26%) had a VQ scan done, none of which were positive for PE. One patient (5.26%) did not have CTPA imaging done.

Our review indicated that 7 of 68 clinicians documented the use of the YEARS algorithm in their work-up. No clinician documented use of the pregnancy-adapted YEARS algorithm. However, 85.1% of the patients evaluated were treated in alignment with the pregnancy-adapted YEARS algorithm. Deviation from the YEARS criteria was observed with seven patients who received unnecessary CTPA imaging and three patients who did not undergo imaging, despite meeting criteria. Two (66.67%) of these three patients met no YEARS criteria and had D-dimer levels >1.0 mg/L, and one patient (33.33%) had one or more YEARS criteria and a D-dimer level of 0.5 mg/L.

**Figure 2.**
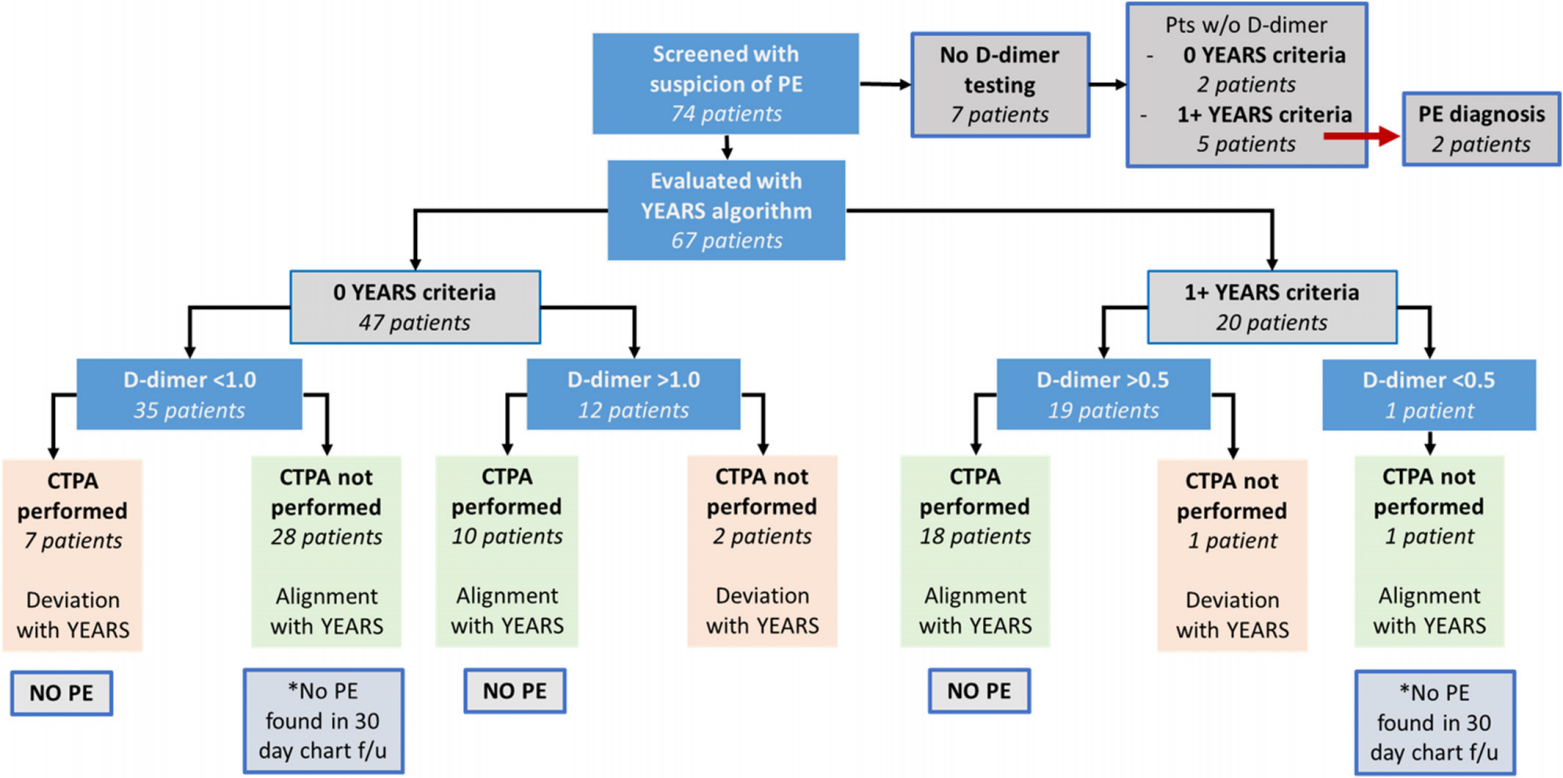
Flow chart of pregnancy-adapted YEARS algorithm in a retrospective diagnostic review. *PE*, pulmonary embolism; *CTPA*, computed tomography pulmonary angiography; *f/u*, follow-up.

The results of the pregnancy-adapted YEARS algorithm applied to our cohort are summarized in Figure 2. Additionally, 14 patients (20.89%) received a lower extremity Doppler, all of which were negative for DVT. Therefore, these patients followed the algorithm outlined in Figure 1. Outcomes of applying the pregnancy-adapted YEARS algorithm to our cohort are summarized in Table 3.

**Table 3.** Outcomes of pregnancy-adapted YEARS algorithm retrospective utilization.

	Total (%)
Patients screened with suspicion of PE (N = 74)	
Patients with PE	2 (2.7%)
Patients with no PE	72 (97.3%)
Patients excluded from YEARS evaluation	7 (9.5%)
Patients available for YEARS evaluation	67 (90.5%)
YEARS algorithm (N = 67)	
Patients treated in concordance to YEARS	57 (85.1%)
Patients not treated in concordance to YEARS	10 (14.9%)
CTPA Use	
Patients who met criteria for CTPA	31 (46.3%)
Patients who did not receive CTPA*	3 (9.6%)
Patients who did not meet criteria for CTPA	36 (53.7%)
Patients who received unnecessary CTPA*	7 (19.4%)
Patients who received a CTPA (or V/Q)	36 (53.4%)
Patients with confirmed pulmonary embolism	2 (5.6%)
Patients with confirmed no pulmonary embolism	34 (94.4%)
Patients who did not receive a CTPA	31 (46.3%)
Failed to receive follow-up	5 (16.1%)
Patients diagnosed with PE or VTE upon 30-day follow-up	0 (0%)

* Patients who were treated non-concordant to the pregnancy-adapted YEARS algorithm.

*PE*, pulmonary embolism; *CTPA*, computed tomography pulmonary angiography; *VTE*, venous thromboembolism; *V/Q*, ventilation/perfusion scan.

## DISCUSSION

In March 2019, the ARTEMIS study was published demonstrating a 39% decrease in CTPA imaging among pregnant patients when using the pregnancy-adapted YEARS criteria.^14^ The ARTEMIS study showed that the pregnancy-adapted YEARS algorithm was able to safely rule out PE in pregnant patients. Following the ARTEMIS study, Langlois et al published a study in May 2019 further applying the pregnancy-adapted YEARS algorithm. This study retrospectively assessed the data from the CT-PE pregnancy study to externally validate the accuracy and safety of the pregnancy-adapted YEARS algorithm. The CT-PE pregnancy study found a 14% decrease in the need for CTPA.^18^ When the pregnancy-adapted YEARS algorithm was retrospectively applied to this data, 32 additional patients had PE excluded without the need for CTPA (78 in total, 21%). This resulted in almost twice as many patients being spared radiation exposure.^18^

The prospective ARTEMIS study and a subsequent retrospective study demonstrated the safety and efficacy of the pregnancy-adapted YEARS algorithm in pregnant patients in a European population. In our study we aimed to conduct an external validation study in the United States of those international studies. In our retrospective study, we found that 36 patients met no criteria to have a CTPA performed, but seven (19.4%) of these patients did receive a CTPA. None of these seven patients had PE detected via the imaging modality. This cohort represents the patients who could have avoided CTPA and radiation exposure if the pregnancy-adapted YEARS algorithm had been applied. Additionally, our cohort consisted of 28 patients who met zero YEARS criteria and had a D-dimer <1.0 mg/L. If a conventional D-dimer cutoff had been used, rather than the algorithm value, our patients would all have had a cutoff value of 0.5 mg/L.^16^ By the intention to diagnose approach, this conventional cutoff would have resulted in an additional 16 patients meeting criteria to undergo CTPA imaging, as 16 of the 28 patients with zero YEARS criteria had a D-dimer level between 0.5–1.0 mg/L.

Combining these with the seven patients who received unnecessary CTPA imaging, our study showed retrospective application of the pregnancy-adapted YEARS algorithm would have resulted in a 34.3% decrease in CTPA utilization. This is consistent with prior prospective studies showing 21% and 32–65% reductions.^14^ ^,^ ^18^ In other words, without actively following the pregnancy-adapted YEARS algorithm, the clinicians who evaluated the patients in our cohort used their clinical judgment to rule out a PE, despite an elevated D-dimer >0.5 mg/L in 16 patients. Given that a substantial percentage (85.1%) of the clinicians evaluated patients in concordance with the pregnancy-adapted YEARS algorithm, our study found that an additional 10.4% of CTPA utilization could have been avoided with active application of the algorithm because 7/67 patients underwent CTPA not in concordance with the algorithm. The ARTEMIS study featured 12 patients (6.2%) who underwent CTPA testing, despite no confirmed DVT and a D-dimer level below the threshold, which was defined as a protocol violation.^14^ Our study showed a similar outcome with seven patients (10.4%) receiving a CTPA despite a D-dimer below the threshold. Therefore, our study validates the current body of research on the YEARS algorithm and the potential utility of the pregnancy-adapted YEARS algorithm in a rural-suburban setting.

Nevertheless, the results from this study have some notable differences compared to recent prospective studies. One difference was the number of patients in our study meeting any YEARS criteria, especially for hemoptysis or clinical signs of a DVT. Among the 67 patients included in the analysis, only 20 patients met one or more YEARS criteria (30%), and of those 20 patients one had unilateral leg swelling and one had both hemoptysis and PE considered as the number one diagnosis. This demonstrates the criterion of PE as the number one diagnosis being the largest contribution in our cohort, resulting in 40/67 (59.7%) patients with a negative YEARS algorithm. This criterion was subject to retrospective bias and may account for variation from previously published prospective studies. Notably, those previous prospective studies showed 49% and 75% of their cohort meeting one or more YEARS criteria.^14^ ^,^ ^18^ Our study additionally featured a smaller sample size than previously published studies, with 67 patients included in the analysis compared to 510 in the ARTEMIS study and and 395 in the Langlois study.^14^ ^,^ ^18^ However, despite our relatively small sample size, we were able to achieve a wide and relatively even spread of gestational ages across all trimesters.

To demonstrate the long-term applicability of the pregnancy-adapted YEARS algorithm, a 30-day chart follow-up was performed on the 36 patients who did not meet criteria for a CTPA. Five of these patients failed to follow up. None of the 31 patients who were reviewed demonstrated evidence of a PE or VTE upon follow-up. This further demonstrates consistency with other studies in the use of the criteria in an acute diagnosis. All patients in the cohort were followed to completion of their pregnancy, and none had a new diagnosis of VTE or an anticoagulant listed on their medication list.

Our study also showed that three of the 31 patients should have received a CTPA according to the pregnancy-adapted YEARS algorithm but did not receive it. These patients are included in the cohort who received treatment that was non-concordant with the algorithm. The first of these patients was a 38-year-old woman in her first trimester with a D-dimer of 1.2 mg/L and no YEARS criteria, who was diagnosed with pneumonia. Literature suggests that pneumonia can cause an elevation of the D-dimer level.^22^ ^,^ ^23^ Pneumonia may present similarly to a PE and represents a diagnosis that could require use of the YEARS algorithm and result in unnecessary CTPA utilization.

The second patient was a 28-year-old woman in her third trimester with a D-dimer of 0.76 mg/L and one YEARS criterion. The evaluating physician used a trimester-adjusted D-dimer and decided that CTPA was not necessary. Literature suggests that D-dimer values fluctuate during pregnancy, and its use alone is not sufficient in ruling out a PE regardless of trimester.^3^ ^,^ ^7^ The third patient was a 28-year-old woman in her third trimester with a D-dimer of 1.48 mg/L with no YEARS criteria. The evaluating physician decided the patient had unspecified dyspnea of unclear origin and ruled that CTPA was not necessary. There were no PE diagnoses for these patients on 30-day follow-up. If counted against the efficacy of pregnancy-adapted YEARS algorithm, additional reduction would decrease from 7/67 (10.4%) to 4/67 (6%), and total reduction with application of the algorithm would decrease to 20/67 (29.85%), which is consistent with prior prospective studies.

Two patients in this study were diagnosed with a PE. Of note, neither of them had a D-dimer completed; therefore, they were excluded from the study. The first patient was 10 weeks pregnant. She presented with chest pain and shoulder pain that increased with inspiration. She had a complex superficial thrombosis of the lower extremity at the time of her workup and was being treated with low molecular weight heparin. Repeat duplex in the ED showed extension of the clot into the deep venous system. The patient’s case was discussed with a maternal fetal medicine physician who recommended CTPA.

The second patient was 33 weeks pregnant. She presented with back pain and was known to be positive for COVID-19 prior to arrival. She also complained of increasing dyspnea and pleuritic chest pain. Given her symptoms and multiple risk factors for clots, the clinician felt that urgent CTPA was necessary. Although these patients were not included in the analysis, they were incorporated into our results for the prevalence of PE during our study period, which was 2.9%. The prevalence of PE in the ARTEMIS study was 5.4%, and in the Langlois study was 6.5%.^14^ ^,^ ^18^ Therefore, our cohort had a lower prevalence of PE compared to the prior European studies. This is also consistent with literature demonstrating the prevalence of ED patients tested for PE in Europe to be 23% compared to 8% in North America.^19^

In total, 57 of the 67 patients (85.1%) in this study were treated in concordance with the pregnancy-adapted YEARS algorithm despite only seven physicians documenting the use of YEARS in their workup. This may indicate that there has already been an informal adoption of the pregnancy-adapted YEARS algorithm in clinical practice. The methodology used by clinicians in the workup of this patient population is similar to the proposed algorithm, which may demonstrate the pregnancy-adapted YEARS algorithm has a higher propensity to be used in clinical practice. However, additional studies are warranted to further elucidate the clinical significance of the pregnancy-adapted YEARS algorithm in different settings and populations Future research should be aimed at demonstrating safety of the algorithm applied to populations in the US.

## LIMITATIONS

This retrospective study is not without its limitations. First, it introduced selection bias in the cohort that was reviewed. The reviewed charts were not originally designed for research; therefore, pertinent information may have been omitted. The criterion of PE as the number one diagnosis falls victim to retrospective bias. Unless explicitly stated, it was subjective in discerning whether the physician believed PE was a primary concern during the medical decision-making process. Another limitation was our small cohort of patients. This may limit the applicability of our results to larger populations. Therefore, the findings and conclusions drawn from this study should be interpreted with caution, recognizing the potential limitations associated with the small sample size. Finally, this study took place in a single health system in northeastern Pennsylvania and may not represent all populations.

## CONCLUSION

Previous prospective studies applying the pregnancy-adapted YEARS algorithm in Europe found 21% and 32-65% reductions in CTPA imaging for pregnant patients with suspected pulmonary embolism.^8^ ^,^ ^18^ Our retrospective study found similar conclusions of the pregnancy-adapted YEARS algorithm. Thus, this study serves as external validation for previous literature in Europe within the United States. Furthermore, this study demonstrated that most clinicians used clinical rationale concordant to the pregnancy-adapted YEARS algorithm, which indicates a potential for widespread adoption for the evaluation of pulmonary embolism in pregnant patients.
